# Supplementary material for: TIMP1 and TIMP2 Downregulate TGFβ Induced Decidual-like Phenotype in Natural Killer Cells
Source: Cancers (Basel). 2021 Oct 1;13(19):4955. doi: 10.3390/cancers13194955 (PMC8507839; doi:10.3390/cancers13194955)
Supplement: Supplementary file 1 [file cancers-13-04955-s001.zip › cancers-1286228-supplementary.pdf]

cancers

Supplementary Materials

TIMP1 and TIMP2 Downregulate TGFβ Induced Decidual-Like Phenotype in Natural Killer Cells

Adriana Albini, Matteo Gallazzi, Maria Teresa Palano, Valentina Carlini, Riccardo Ricotta, Antonino Bruno, William G. Stetler-Stevenson and Douglas M. Noonan

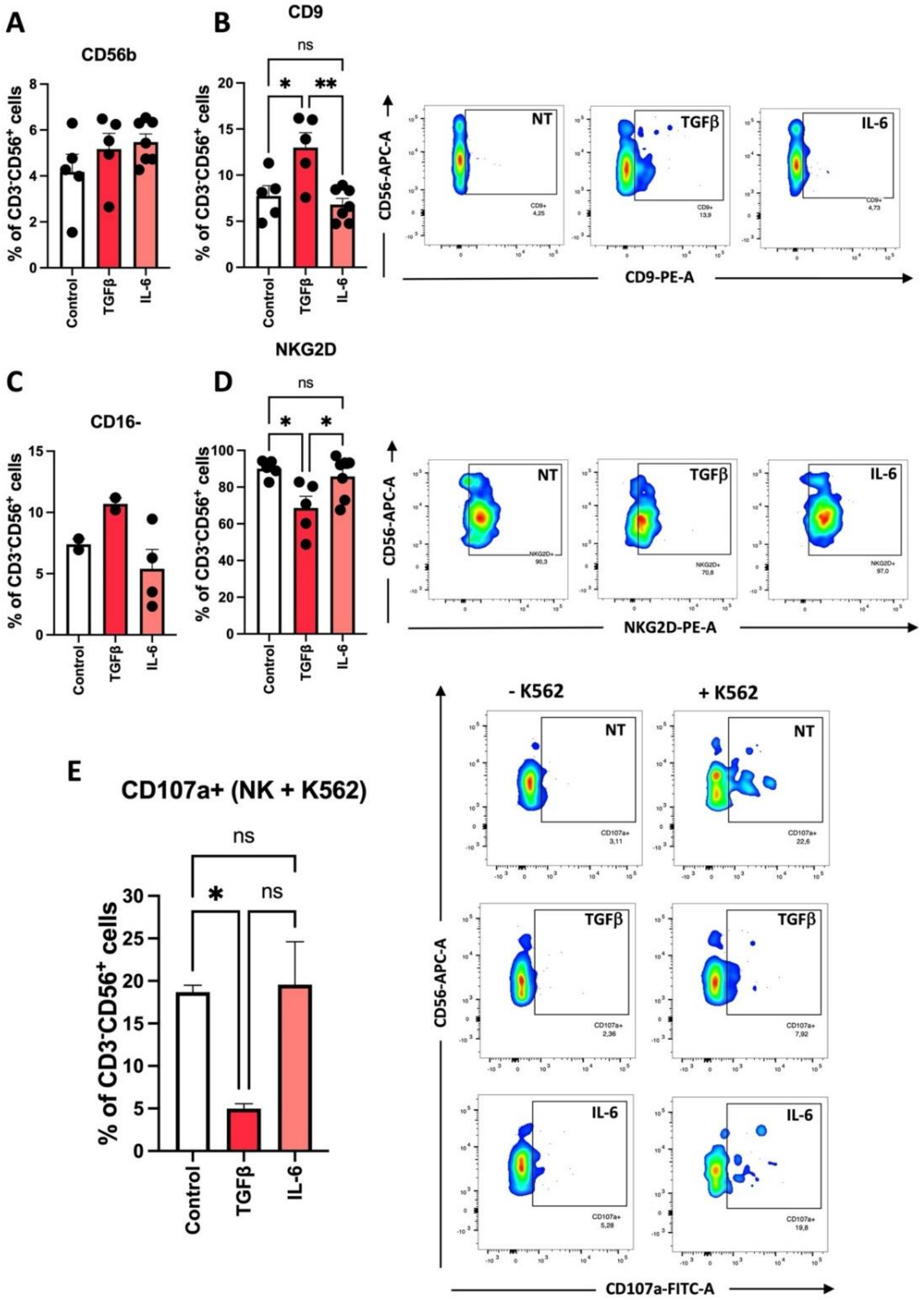

Supplementary Figure S1. Comparison of the effects of TGFβ and IL-6 in generating of decidual-like/anergic/exhausted NK cells. We observed no differences in CD56^{bright} NK cell increase when compared TGFβ with IL-6 (A), while NK cells exposed to TGFβ has increased expression of CD9, compared with not treated and IL-6 exposed NK cells (B). Also, TGFβ was more effective than IL-6 in increasing the frequency of CD16 NK cells (C), and in

downregulating NKG2D (D). TGFβ significantly reduced NK cell degranulation capabilities against K562 cells, as compared to IL-6 (E). Results are showed mean ± SEM, ANOVA, ns= not signficant; *p < 0.05; **p < 0.01. Controls were cells in RPMI medium alone. PBMCs from 2-7 different healthy donors were used.

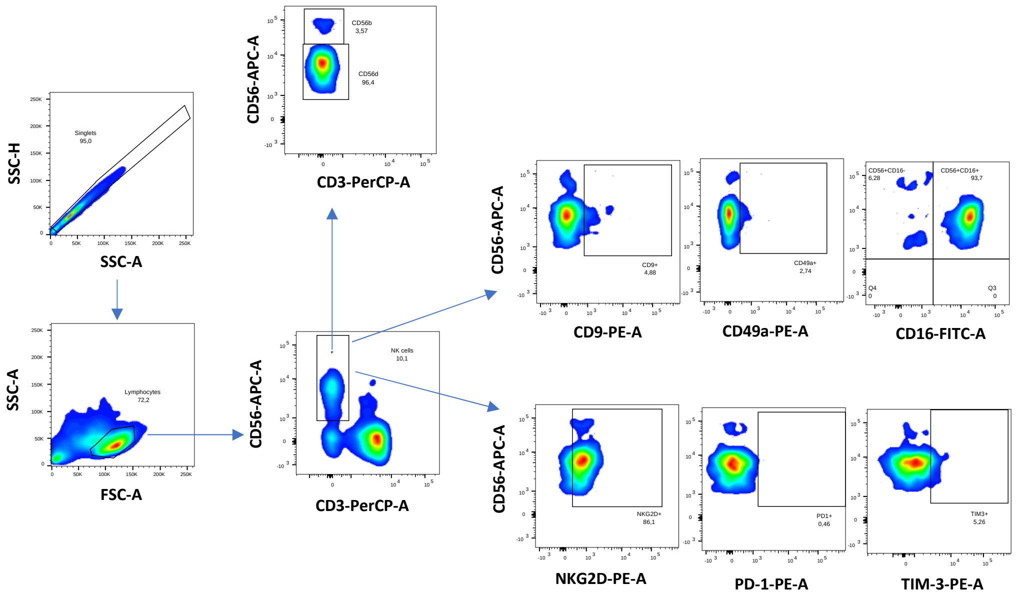

Supplementary Figure S2. Representative gating strategy for NK cell identification and selected decidual-like /CD56bright, CD9, CD49a), activation (NKG2D) and exhaustion (PD-1, TIM-3) detection.

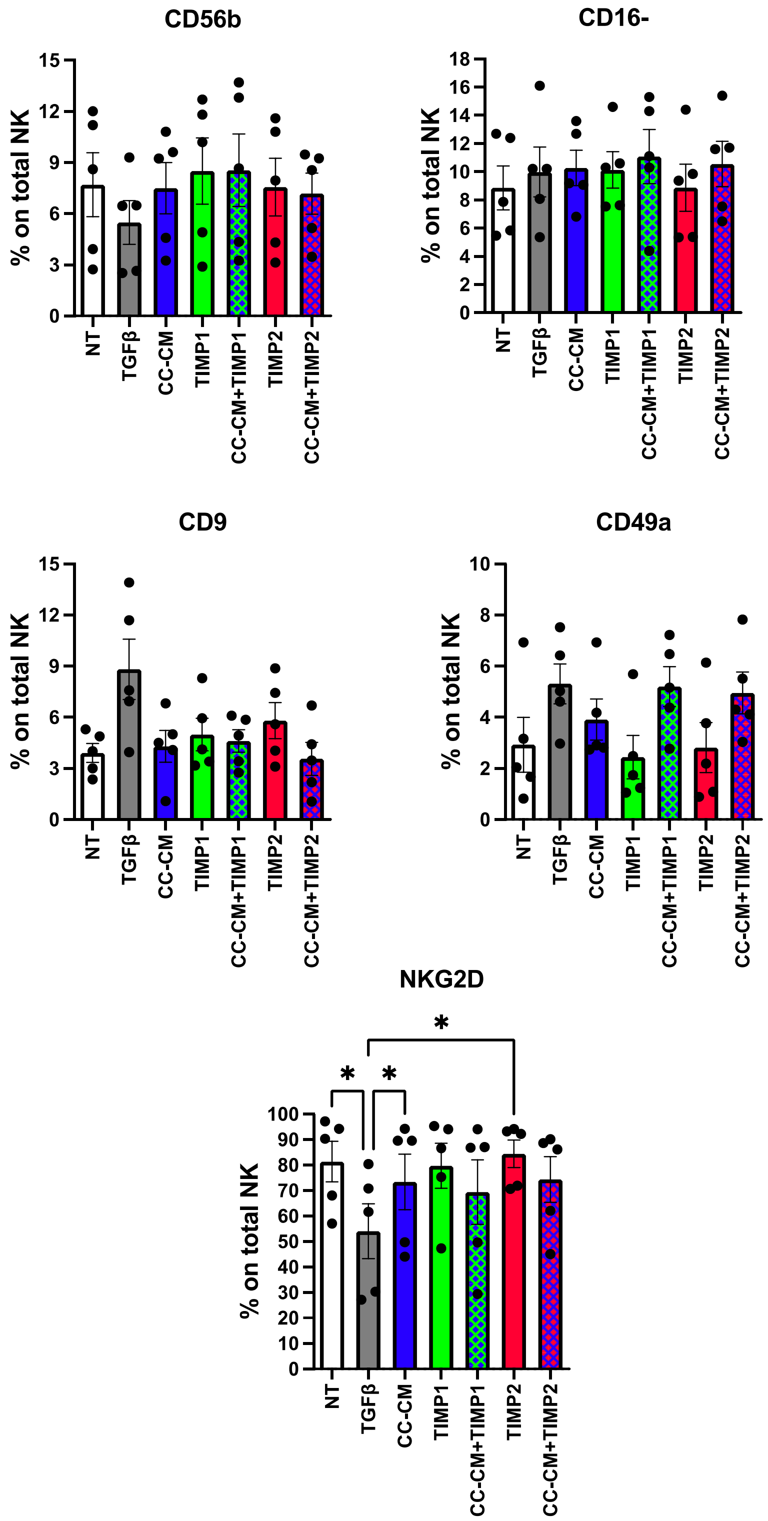

Supplementary Figure S3. The CM of CC and CRC cell lines increase the surface expression CD49a, NKG2D and TIM-3 levels, but not the CD56^{bright}, CD16^{neg} and CD9. TIMP-1 and TIMP-2 treatments showed a trend in being effective in regulating divergent CD49a, NKG2D and TIM-3 exhaustion marker. Results are showed as mean ± SEM. PBMCs of 3 to 5 healthy donors were used.

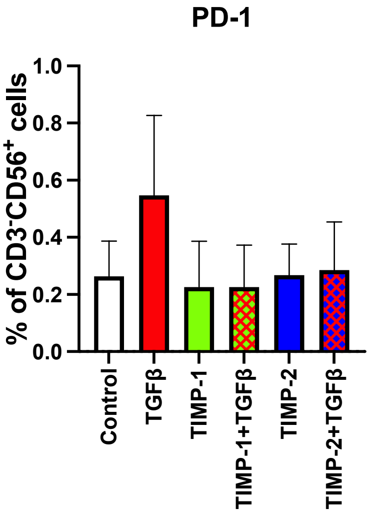

Supplementary Figure S4. TIMP-1 and TIMP-2 treatments showed a trend in contrasting the TGFβ-induced ability to increase PD-1 expression in cytolytic NK cells. PBMCs of 6 healthy donors were used. Results are showed as mean ± SEM.

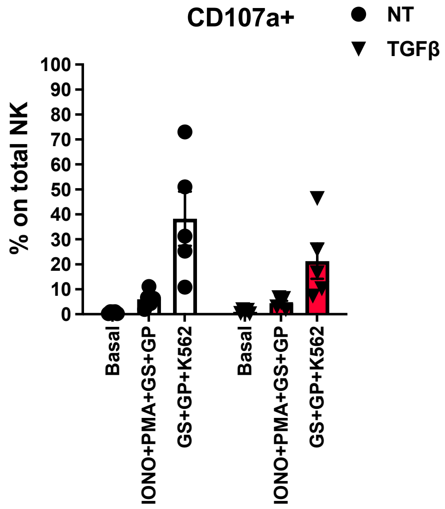

Supplementary Figure S5. Internal controls for NK cell degranulation capability. The graph shows the degranulation capabilities of control and TGFβ treated NK cells at basal level (NK only in RMPI complete medium), NK cells with induced non-specific degranulation (NK only in RPMI complete medium, supplemented with Ionomycin, PMA, BrefeldinA and Monensin) and NKs with specific degranulation (NK cells and K562 cells at 1:1 effector/target ratio, in RPMI complete medium, supplemented with BrefeldinA and Monensin). Results are showed as mean ± SEM.
